# Genomic surveillance of influenza A virus in live bird markets during the COVID-19 pandemic

**DOI:** 10.14202/vetworld.2025.955-968

**Published:** 2025-04-23

**Authors:** Ni Luh Putu Indi Dharmayanti, Diana Nurjanah, Risa Indriani, Teguh Suyatno, Harimurti Nuradji

**Affiliations:** 1 Research Organization for Health, National Research and Innovation Agency, Cibinong, 16911, Indonesia; 2 Research Centre for Veterinary Science, Research Organization for Health, National Research and Innovation Agency, Cibinong, 16911, Indonesia

**Keywords:** coronavirus disease 2019, genomic surveillance, H9N2, influenza A virus, live bird markets, reassortment, zoonosis

## Abstract

**Background and Aim:**

Despite the global focus on coronavirus disease 2019 (COVID-19), the avian influenza virus (AIV) continues to circulate in Indonesia, particularly in traditional live bird markets (LBMs), which serve as critical nodes for virus amplification and interspecies transmission. This study aimed to investigate the co-circulation and genetic features of AIV, particularly the H9N2 subtype, and severe acute respiratory syndrome coronavirus 2 (SARS-CoV-2) in LBMs in East Java during the COVID-19 pandemic.

**Materials and Methods:**

Environmental surveillance was conducted in seven traditional markets across four districts in East Java Province in 2021. Surface swabs were collected from high-risk areas, including poultry display tables, knives, cutting boards, and napkins. Samples were tested using reverse transcriptase polymerase chain reaction for influenza A and SARS-CoV-2. Positive AIV samples were further subtyped, sequenced, and analyzed for genetic markers associated with virulence, reassortment, and mammalian adaptation.

**Results:**

Of 156 samples tested, 17 (10.9%) were positive for influenza A, with 3 (1.9%) confirmed as the H9 subtype. These H9-positive samples were collected from a knife, cutting board, and napkin in the same market location in Lamongan Regency. Phylogenetic and molecular analyses revealed that two isolates (LSJ/Env/83 and LSJ/Env/84) were H9N2 reassortants, harboring key molecular markers such as Q226L, T160A, and S138A in the hemagglutinin protein, indicative of increased affinity for human-type receptors. Additional substitutions in PB2, MP, and NS1 proteins were associated with enhanced replication and virulence in mammalian and avian hosts. All samples tested negative for SARS-CoV-2.

**Conclusion:**

This study demonstrates the continued environmental circulation of reassortant H9N2 AIVs in traditional markets during the COVID-19 pandemic, with isolates displaying genetic features indicative of zoonotic potential. These findings underscore the necessity for sustained genomic surveillance and stricter biosecurity interventions in LBMs to prevent cross-species transmission and mitigate pandemic risk.

## INTRODUCTION

During the coronavirus disease 2019 (COVID-19) pandemic, data from Indonesia’s Animal Health Information System (https://validation.isikhnas.com) indicated a decline in reported cases of highly pathogenic avian influenza (HPAI) and low pathogenic avian influenza (LPAI) in birds from 2021 to 2023. Nonetheless, both HPAI and LPAI continued to be detected not only in animals but also in the environment [1, 2]. This persistent detection suggests that the actual extent of avian influenza virus (AIV) transmission in the field may be underestimated, likely due to ongoing environmental contamination.

Traditional or live bird markets (LBMs), characterized by high poultry density and species diversity across various age groups, serve as critical hotspots for interspecies transmission due to the continuous circulation and amplification of different AIV strains [3, 4]. Shi *et al*. [5] have demonstrated that zoonotic influenza outbreaks are frequently initiated by the sustained presence of the virus within live poultry markets. Given the potential for novel influenza A virus strains to circulate in such settings, these markets are considered to be significant contributors to the emergence of zoonotic influenza infections and represent a potential trigger for future pandemics. Historical examples include human infections with zoonotic viruses such as H7N9 in China (2013), H9N2 in China (2021), and H5N1 in China (2008–2009), all linked to live poultry market exposure [6–9].

In Indonesia, the prevalence of HPAI and LPAI in poultry has fluctuated over time, although a general declining trend is observed. Despite this, AIV continues to evolve silently through genetic reassortment, resul- ting in the emergence of novel strains and raising concerns about the possibility of a future pandemic [10]. Moreover, these reassortant viruses may escape detection by pre-existing neutralizing antibodies in the broader population, thereby increasing the risk of outbreaks in both humans and animals. Certain reassortant strains have demonstrated heightened pathogenicity, increased virulence, and enhanced transmission capabilities compared to their progenitors [11–13]. Yan and Wu [14], Park *et al*. [15], and Choudhury *et al*. [16] have also shown that such reassortant viruses can cross species barriers, enabling infection of new host species. Evidence suggests that reassortant strains are likely to dominate the future evolutionary landscape of influenza viruses, representing a substantial risk for potential outbreaks [17, 18].

Although previous studies have documented the circulation of AIV, including reassortant strains, in live poultry markets across Indonesia, comprehensive environmental surveillance data during the COVID-19 pandemic remain limited. Most available reports have focused primarily on AIV detection in poultry rather than evaluating environmental contamination, which plays a critical role in sustaining virus transmission. Furthermore, there is a paucity of genomic characterization of reassortant influenza A viruses isolated from environmental sources in traditional markets, particularly regarding their potential for cross-species transmission. The interaction between ongoing viral evolution, such as reassortment events, and market-based risk factors during a concurrent global health crisis (i.e., COVID-19) has not been adequately investigated. This knowledge gap hinders the early identification of emerging zoonotic threats and the development of targeted mitigation strategies.

This study aimed to investigate the circulation and genetic characteristics of influenza A viruses, including potential reassortants, in environmental samples collected from traditional LBMs in East Java, Indonesia, during the COVID-19 pandemic. In particular, the study sought to (1) detect and subtype influenza A viruses and severe acute respiratory syndrome coronavirus 2 (SARS-CoV-2) from environmental surfaces, (2) characterize the molecular features of detected AIVs with a focus on markers associated with mammalian adaptation and virulence, and (3) assess the implications of these findings for zoonotic transmission and public health surveillance.

## MATERIALS AND METHODS

### Ethical approval

Ethical approval for the study was granted by the Indonesian Agency for Agricultural Research and Development and the Institutional Animal Care and Use Committee under registration number Balitbangtan/BB Litvet/M/01/2021, ensuring all procedures adhered to established ethical guidelines.

### Study period and location

This study was conducted in 2021 and focused on traditional live bird markets (LBMs) across four regions within East Java Province, Indonesia. Environmental surveillance activities were carried out in seven markets, specifically two markets each in Surabaya City, Sidoarjo Regency, and Gresik Regency, as well as one market in Lamongan Regency.

### Sample collection

Surveillance activities were carried out in several traditional markets located across various regencies and a city in East Java Province in 2021. These included two markets each in Surabaya City, Sidoarjo Regency, and Gresik Regency and one market in Lamongan Regency. These sites were selected based on their high density of poultry-related activities, the diversity of market types, and the wide range of socioeconomic and geographical conditions. By including both urban and peri-urban areas, the study aimed to offer a comprehensive assessment of AIV circulation across diverse settings, where risk factors for zoonotic transmission - such as population density, market infrastructure, and human-animal interactions - may differ.

**Figure 1:**
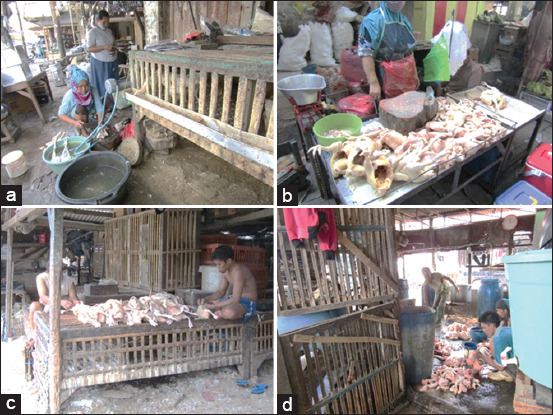
Environmental sampling sites in traditional markets during the coronavirus disease 2019 pandemic. (a) Traders engage in slaughtering, washing, and cutting poultry in close proximity to live birds, often without appropriate personal protective equipment (PPE), increasing the risk of viral transmission. (b) Chicken meat display tables and surrounding objects in traditional markets, which may act as potential sources of viral contamination. (c) Poultry feather plucking is performed without proper PPE and in close proximity to live birds, raising concerns about biosecurity and disease transmission. (d) Poultry carcass washing occurs directly on the floor without PPE, posing a risk of viral contamination and environmental exposure.

Sampling focused on objects with frequent contact with poultry, including live birds, poultry products, and slaughtered poultry (Figure 1). Surface swabs were collected from chicken vendor stalls, targeting locations such as carcass display tables, knives, napkins, drainage or gutter areas, floors or the surrounding ground, poultry washing tanks, and weighing scales.

Samples were transported in a portable freezer maintained at −20°C and then stored at −80°C until testing. Laboratory analysis was conducted using reverse transcriptase polymerase chain reaction (RT-PCR) at the Virology Laboratory of the Center for Veterinary Instrument Standard Testing (BBPSI Veteriner), Bogor and the Genomic Laboratory of the National Research and Innovation Agency in Cibinong. Positive samples were propagated in specific pathogen-free embryonated chicken eggs (9–11 days old) sourced from PT. Caprifarmindo Laboratories, Bandung, Indonesia.

### RT-PCR and partial DNA sequencing

Viral RNA was extracted from infected allantoic fluid using the QIAamp RNA Mini Kit (Qiagen), following the manufacturer’s instructions. The extracted RNA was tested for influenza A subtypes H5, H9, and H3 using RT-PCR with specific primers. DNA sequencing was subsequently conducted on influenza A-positive samples based on the protocols described by Dharmayanti *et al*. [19–21]. In parallel, detection of SARS-CoV-2 was performed using the SensiFAST^™^ Real-Time PCR Kits (Bioline, Meridian BioScience, Ohio, USA) targeting the N1, N2, and RP genes, in accordance with the manufacturer’s instructions.

Sequencing results were verified and edited using BioEdit v7.7.1.0 (https://thalljiscience.github.io/). Phylogenetic trees were constructed using the maximum likelihood method based on the Tamura-Nei model in Molecular Evolutionary Genetics Analysis 7 software (https://www.megasoftware.net/). The robustness of the phylogenetic analysis was assessed through bootstrap testing with 1,000 replicates. Viral genetic data were then compared with approximately 50–100 nucleotide and amino acid sequences obtained from the National Center for Biotechnology Information database. This bioinformatic analysis aimed to identify AIV mutations associated with reassortment, antigenic shift, or antigenic drift. The resulting DNA sequences were submitted to the GISAID database (https://gisaid.org/) under the following accession numbers: EPI2626726, EPI2626727, EPI2626725, EPI2626729, EPI2626722, EPI2626728, EPI2626724, EPI2626723, EPI2626734, EPI2626735, EPI2626733, EPI2626737, EPI2626730, EPI2626736, EPI2626732, and EPI2626731 for the isolates LSJ/Env/83 and LSJ/Env/84.

## RESULTS

**Figure 2:**
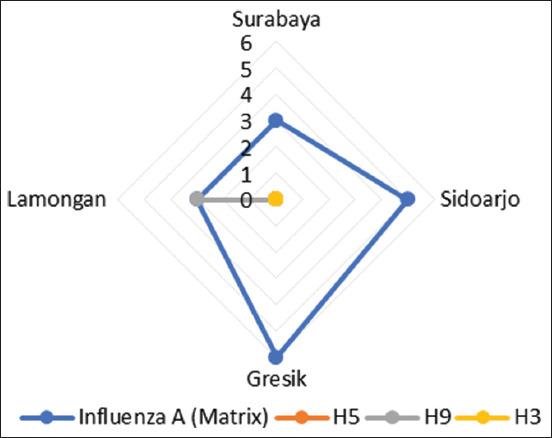
Distribution of influenza A (matrix [M] gene) and subtype detections across different locations. The radar chart illustrates the number of positive samples for the influenza A virus (M gene) and its subtypes (H5, H9, and H3) in four regions: Surabaya, Sidoarjo, Gresik, and Lamongan. Influenza A (M gene) was predominantly detected in Gresik (n = 6) and Sidoarjo (n = 5), with lower detections in Surabaya (n = 3) and Lamongan (n = 3). In addition, three H9 subtypes were identified in the Lamongan regency. These findings suggest spatial variability in the prevalence of different influenza A subtypes within the surveyed locations.

Based on the surveillance data, 17/156 tested samples (10.9%) were positive for influenza A virus, with positive detections distributed across the city and regencies. Specifically, three samples (1.92%) were identified in Surabaya City, five (3.2%) in Sidoarjo Regency, six (3.84%) in Gresik Regency, and three (1.92%) in Lamongan Regency. The influenza A-positive samples, which tested positive for the matrix (M) gene, were further subtyped for the H5, H9, and H3 genes. Of the 17 M gene-positive samples, three (17.6%) were positive for the H9 gene (Figure 2), and all were negative for the H5 and H3 subtypes. Notably, all H9-positive samples originated from the same market in Lamongan Regency, suggesting the presence of localized viral activity. This observation raises the possibility that this site may serve as a potential hotspot for AIV transmission, possibly influenced by specific environmental or trade-related factors that support viral persistence and spread. In addition to the current findings, a previous study by Dharmayanti *et al*. [19] reported the presence of H5 in poultry and environmental contamination by H5 AIV in markets within Lamongan, further emphasizing this location as a potential focal point for the transmission of multiple AIV subtypes.

All samples tested negative for SARS-CoV-2 during the surveillance period. This absence may be attributed to various factors, including temporary market closures, the specific timing of sample collection, and potential limitations in assay sensitivity. Furthermore, environmental instability, airborne transmission characteristics, and sampling strategy limitations may have also contributed to the lack of SARS-CoV-2 detection in these market settings. In contrast to zoonotic AIV subtypes such as H9, SARS-CoV-2 has not demonstrated effective infectivity or replication in poultry species [22, 23]. Therefore, if birds do not serve as natural hosts or reservoirs, the likelihood of environmental detection is low. This is further supported by the fact that SARS-CoV-2 primarily spreads through aerosols and respiratory droplets, whereas surface contamination plays a minimal role in transmission [24]. These factors, collectively limiting viral stability on surfaces, may reduce the probability of detecting SARS-CoV-2 in environmental samples from traditional markets. Further investigation is warranted to assess the extent to which these factors influenced the observed results.

**Figure 3:**
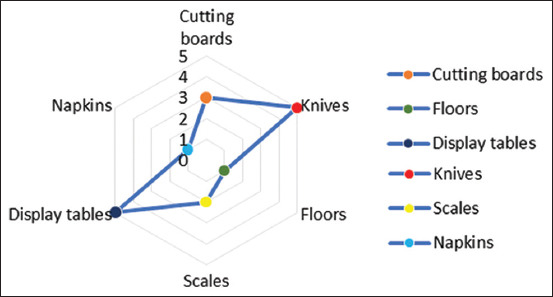
Distribution of influenza A virus (matrix gene) detection across different environmental surfaces. The radar chart illustrates the number of positive samples detected on various surfaces associated with poultry handling, including knives, display tables, cutting boards, napkins, scales, and floors. The highest number of positive samples was found on knives and display tables (n = 5 each), followed by cutting boards (n = 3), scales (n = 2), and napkins and floors (n = 1 each). These findings highlight the potential high-risk areas for viral contamination in poultry markets.

**Figure 4:**
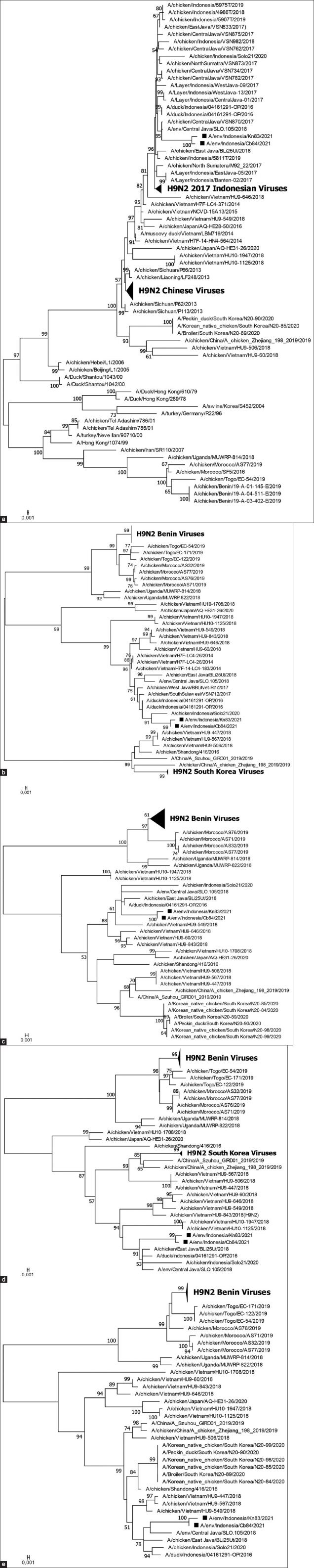
Phylogenetic trees of (a) hemagglutinin; (b) neuraminidase; (c) matrix protein; (d) nucleoprotein; and (e) non-structural protein genes of influenza A H9N2 viruses that were isolated from this study. The viruses are shown as black square marks.

At multiple market sampling points associated with live poultry, poultry products, and slaughtering activities – such as display tables, knives, cutting boards, and washing tanks – the M gene of influenza A virus was most frequently detected on display tables and knives, with five positive samples each (3.2%). Based on these findings, three positive samples (1.92%) were identified on cutting boards, 2 (1.28%) on weighing scales, and 1 (0.64%) each on floors and napkins (Figure 3). In addition, three samples tested positive for the H9 gene subtype - LSJ/Env/83, LSJ/Env/84, and LSJ/Env/100 - collected from a knife, cutting board, and napkin, respectively. Two of these samples, LSJ/Env/83 (designated A/Env/Indonesia/Kn83/21) and LSJ/Env/84 (designated A/Env/Indonesia/Cb84/21), were successfully sequenced and originated from swabs taken from a knife and cutting board used by poultry meat vendors. Phylogenetic analysis of the hemagglutinin (HA), neuraminidase (NA), M, non-structural protein (NS), and nucleoprotein genes indicated that both isolates belong to the H9N2 subtype (Figure 4). Interestingly, Basic Local Alignment Search Tool analysis of other viral gene segments suggested that they belong to an HxNx subtype (https://blast.ncbi.nlm.nih.gov/Blast.cgi).

Genetic analysis of the HA protein in LSJ/Env/83 revealed amino acid substitutions at positions 155, 183, 226, 138, 160, 159, 190, and 198 - markers known to be associated with increased affinity for human receptors [25]. In comparison, LSJ/Env/84 exhibited all of these substitutions except for the A190 mutation (Table 1). These findings underscore the presence of molecular features in the HA protein that enhances binding to human-type receptors, potentially elevating the risk of zoonotic transmission. When compared to previously characterized strains, both LSJ/Env/83 and LSJ/Env/84 contain critical substitutions - such as Q226L - associated with enhanced binding to human-like receptors [26]. Additional substitutions, including T160A and S138A, further support the likelihood of increased interspecies transmission potential [27, 28].

**Table 1:** Amino acid substitution in HA protein to predict its affinity to human-type receptors.

Viruses/Isolates name	Increased affinity for human-type receptors (H3 numbering)							

	I155T	H183N	A190T/V	Q226L	S138A	T160A	S159N	N198T
LSJ/Env/83	T	N	V	L	A	A	N	T
LSJ/Env/84	T	N	A	L	A	A	N	T
A/turkey/Wisconsin/1/1966	T	H	-	Q	A	-	N	-
A/chicken/Beijing/1/94	T	N	V	Q	A	A	N	T
A/chicken/Guang dong/CJS01, 2013.	T	N	V	L	A	A	N	T
A/muscovy duck/Vietnam/LBM719/2014	T	N	A	L	A	A	N	T
A/Layer/Indonesia/Banten-01/2017	T	N	A	L	A	A	N	T
A/Quail/Hong Kong/G1/1997	T	H	-	L	A	-	-	N
A/chicken/Republic of Korea/AI-96004/1996	T	H	-	Q	A	-	-	-
A/duck/Hong Kong/Y280/97	T	H	-	Q	A	-	N	-
A/duck/Hong Kong/Y439/1997	T	N	T	L	A	A	N	T
A/Guangdong/W1/2004 (human H9N2)	T	N	V	L	A	T	N	T
A/Hubei/1187/2019 (human H9N2)	T	N	A	L	A	A	N	T

HA=Hemagglutinin

For other proteins, including polymerase basic protein 2 (PB2), M protein (MP), and NS, both isolates contained all relevant adaptation markers, with the exception of PB2 A588V, which was only present in LSJ/Env/84 (Table 2). These results highlight molecular features that support the adaptation of H9N2 AIV to mammalian hosts. Specific mutations in the PB2, M1, and NS1 genes are known to enhance viral replication efficiency and immune evasion in mammalian systems [29–31]. Notably, the PB2-588V mutation in LSJ/Env/84 has previously been linked to increased polymerase activity in mammalian cells [32]. Similarly, the M1-30D [33] and NS1-138F [34] mutations - present in both isolates - have been associated with increased virulence in mammalian hosts. These molecular markers distinguish the current isolates from earlier avian strains and emphasize their potential for cross-species transmission and increased pathogenicity in mammals.

**Table 2:** Molecular determinants of AIV H9N2 adaptation in mammalian hosts.

Protein names	Residue	Avian-like motif	Mammalian-like Motif	LSJ/Env/83	LSJ/Env/84	A/chicken/Sidrap/07161511–057/2016
PB2	504	I	V	V	Not detected	V
	588	A	V	A	A	V
M1	30	N	D	D	D	D
	43	I	M	M	M	M
	215	T	A	A	A	A
NS1	42	P	S	S	S	S
	138	C	F	F	F	F
	149	V	A	A	A	A

AIV=Avian influenza virus, PB2=Polymerase basic protein 2, NS1=Non-structural protein 1, M1=Matrix 1

A comparative analysis of the HA protein’s molecular features in the current H9N2 isolates and those of other lineages is provided in Table 3. Both LSJ/Env/83 and LSJ/Env/84 share conserved characteristics, including the NSTE glycosylation site, NGLC receptor-binding pocket, and NVSY motif. These features are common in AIV but also found in certain human H9N2 strains such as A/Hubei/1187/2019. A notable distinction between LSJ/Env/83 and LSJ/Env/84 lies in the cleavage site motif: LSJ/Env/83 exhibits PSRSSRGLF, identical to the human H9N2 A/Hubei/1187/2019, whereas LSJ/Env/84 shows a slight variation (PSKSSRGLF). Such differences may influence replication efficiency and host adaptation. Compared to human H9N2 strains such as A/Guangdong/W1/2004 and A/Hubei/1187/2019, both isolates display similar antigenic sites - particularly at GTSKA and NGLMGR - suggesting a degree of antigenic drift toward human adaptation. However, unlike typical human-adapted strains, which predominantly contain NVTY in the receptor-binding pocket, both isolates in this study retain the NVSY motif, more characteristic of avian strains. This indicates that while LSJ/Env/83 and LSJ/Env/84 possess several molecular features conducive to mammalian adaptation, additional mutations are likely required for full adaptation to human hosts.

**Table 3:** Comparison of the molecular characteristics of the HA protein of AIV subtype H9N2 virus in this study.

Isolate name	Lineage	Potential glycosylation sites							Cleavage site	Receptor-binding pocket			Antigenic site	
			
		11–14	87–90	123–126	200–202	280–283	287–290	295–297	315–323	Binding site	Right side	Left side	Site 1	Site 2
				
										92, 143, 145, 173, 180, 184, and 185	128–132	214–219	125, 147, 152	135, 183, 216
LSJ/Env/83	Not determine	NSTE	NGLC	NVSY	NRT	NTTL	NVSK	NCS	PSRSSRGLF	PWTNVLY	GTSKA	NGLMGR	SKP	DNL
LSJ/Env/84	Not determine	NSTE	NGLC	NVSY	NRT	NTTL	NVSK	NCS	PSKSSRGLF	PWTNALY	GTSKA	NGLMGR	SKP	DNL
A/turkey/Wisconsin/1/1966	American	NSTE	NGMC	NVTY	NRT	NTTL	NISK	NCPK	PAVSSRGLF	PWTHELY	GTSRA	NGQQGR	TKP	NDQ
A/chicken/Beijing/1/94	BJ 94	NSTE	NGMC	NVTY	NRT	NTTL	NVSK	NCPK	PARSSRGLF	PWTNVLY	GTSRA	NGQQGR	TKP	DNQ
A/chicken/Guang dong/CJS01, 2013.	CVI	NSTE	NGLC	NVSY	NRI	NTTL	NVSK	NCSK	PSRSSRGLF	PWTNVLY	GTSKA	NGLMGR	SKP	DNL
A/muscovy duck/Vietnam/LBM719/2014	CVI	NSTE	NGLC	NVSY	NRT	NTTL	NVSK	NCSK	PSRSSRGLF	PWTNALY	GTSKA	NGLMGR	SKP	DNL
A/Layer/Indonesia/Banten-01/2017	CVI	NSTE	NGLC	NVSY	NRT	NTTL	NVSK	NCSK	PSKSSRGLF	PWTNALY	GTSKA	NGLMGR	SKP	DNL
A/Quail/Hong Kong/G1/1997	G1	NSTE	NGTC	NVTY	NRT	NSTL	NISK	TCPK	PARSSRGLF	PWTHELY	GTSKA	NGLMGR	TKP	GNL
A/chicken/Republic of Korea/AI-96004/1996	Korean	NSTE	NGMC	NVTY	NRT	NTTL	NVSK	NCPK	PAASYRGLF	PWTHELY	GISRA	NDLQGR	TKP	NNQ
A/duck/Hong Kong/Y280/97	Y280	NSTE	NGLC	NVSY	NRT	NTTL	NVSK	NCPK	PARSSRGLF	PWTNTLY	GTSKA	NGQQGR	SKP	DNL
A/duck/Hong Kong/Y439/1997	Y439	NSTE	NGMC	NVTY	NRT	NTTL	NVSK	NCPK	PAASNRGLF	PWTHELY	GTSKA	NGLQGR	TKP	NNQ
A/Guangdong/W1/2004 (human H9N2)	BJ 94	NSTE	NGMC	NVTY	NRA	NTTL	NVSK	NCPK	PARSSRGLF	PWTNVLY	GTSSA	NGLQGR	TKP	NNL
A/Hubei/1187/2019 (human H9N2)	Y280	NSTE	NGLC	NVSY	NRI	NTTL	NVSK	NCSK	PSRSSRGLF	PWTNALY	GTSKA	NGLMGR	SKP	DNL

AIV=Avian influenza virus, LBMs=Live bird markets, HA=Hemagglutinin

**Figure 5:**
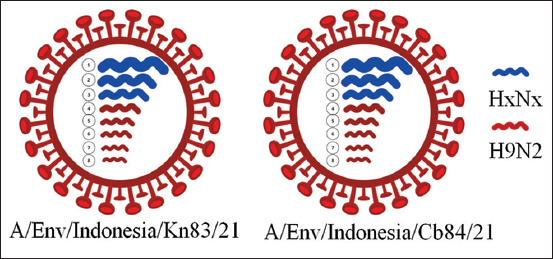
The gene constellation of the reassortant H9N2 viruses isolated from environmental contamination in critical areas of live bird markets in East Java, Indonesia (1) polymerase basic protein 2 gene, (2) polymerase basic protein 1 gene, (3) polymerase acidic gene, (4) hemagglu-tinin gene, (5) nucleoprotein gene, (6) neuraminidase gene, (7) matrix protein gene, and (8) non-structural protein gene.

Figure 5 schematically represents the reassortant H9N2 virus. The virus was detected on a knife, cutting board, and napkin, all collected from the same market in Lamongan Regency, East Java Province. These results suggest that H9N2 virus circulation within the market environment may contribute to ongoing environmental contamination, underscoring the risk of viral persistence in poultry trade settings.

## DISCUSSION

### The role of traditional poultry markets as hubs for viral transmission

Traditional markets in Indonesia, present in both rural and urban areas, serve as central points for the sale of various goods, including live poultry. These birds are sometimes slaughtered on-site within the market premises. Poultry in these settings varies in origin, age, and species and is often kept in close proximity, creating ideal conditions for cross-species transmission. Such environments facilitate co-infection and reassortment of AIV, potentially giving rise to novel strains with enhanced transmissibility to mammals and humans. In addition, waste products from poultry slaughtering activities - such as blood, body fluids, and feathers - may further contribute to viral dissemination.

### Circulation of influenza A virus and environmental contamination pathways

The findings of this study revealed that multiple critical areas within poultry trading zones and their surrounding environments were contaminated with the influenza A virus. Dharmayanti *et al*. [19] reported that the H5N1 subtype circulated in live animal markets across Banten, Central Java, East Java, Jakarta, and West Java between 2014 and 2019. Notably, reassortant viruses were also isolated from environmental samples in Surakarta City in 2014 [19]. Consistent with those findings, the current study showed that the highest levels of contamination occurred on surfaces around butchering and meat-selling areas, namely display tables (5%), knives (5%), cutting boards (1.92%), scales (1.28%), and floors and napkins (0.64%).

Contamination in these areas likely resulted from handling infected poultry, especially when the infection was undetected, allowing meat and internal organs to harbor the virus and contribute to its environmental circulation. Among the objects sampled, display tables had the highest contamination, possibly due to their prolonged contact with poultry carcasses compared to other surfaces with less direct exposure.

Since 2016, the H9N2 AIV has been reported in Indonesia and remains in circulation [35]. According to the Indonesian Ministry of Agriculture’s iSIKHNAS System (https://validation.isikhnas.com/), AIV continues to affect poultry populations in several provinces. Although HPAI and LPAI cases have fluctuated, a decline in LPAI cases - including H9N2 - was observed in 2022 (n = 260) and 2023 (n = 10), compared to 2020 (n = 508) and 2021 (n = 1,056). However, these data were limited from 2020 onward, and information from earlier years remains unavailable. A study by Parums [10] also reported a 99% reduction in influenza diagnoses during the COVID-19 pandemic, aligning with global declines in influenza-related mortality in 2020.

This downward trend may be attributable to public health measures such as social distancing during the pandemic. Nonetheless, despite the reported reduction in LPAI cases, this study detected H9N2 reassortant viruses in traditional market environments. These findings suggest potential gaps in current surveillance or the presence of unique viral traits that enable undetected circulation. Furthermore, LPAI H3 subtype viruses were identified in East Java markets during 2021 surveillance efforts [10]. These observations imply that AIV mutations continue silently, posing a possible risk for future pandemics driven by emerging strains [36, 37].

### Adaptation of the H9N2 virus to mammalian hosts

To explore the characteristics of the H9N2 virus isolated in this study, molecular markers linked to mammalian adaptation were examined. Isolate LSJ/Env/83 contained several substitutions associated with enhanced affinity for human-like receptors, including I155T, H183N, A190V, Q226L, S138A, S159N, N198T, and T160A. Meanwhile, LSJ/Env/84 exhibited seven of these eight substitutions, lacking only A190V [38].

Mutations at position 226 in the receptor-binding site (RBS) of the HA protein, particularly the L226 variant, enhance binding to α2,6-linked sialic acid receptors common in humans, whereas Q226 favors binding to α2,3-linked receptors found in birds [39]. Studies have also shown that L226 and T198 mutations promote virus replication and contact transmission in ferrets. The V190 mutation similarly enhances receptor binding and viral replication in mammalian cells [40]. The A190V substitution was present in LSJ/Env/83 but not in LSJ/Env/84 or A/Layer/Indonesia/Banten-01/2017. Both LSJ/Env/83 and LSJ/Env/84 carried other critical markers in PB2, MP, and NS genes, except for PB2 A588V. These markers have been associated with increased virulence in mice, elevated polymerase activity, replication in mammalian and avian cell lines, greater virulence in chickens and ducks, and suppression of interferon responses [40].

Both isolates exhibited typical LPAI cleavage site motifs – RSSR↓GLF in LSJ/Env/83 and KSSR↓GLF in LSJ/Env/84 - recognized by trypsin-like proteases in the respiratory and digestive tracts, thereby restricting replication to these tissues [41, 42].

In addition, both isolates carried the A316S substitution at the cleavage site, a mutation known to enhance virulence in chickens. This substitution increases HA cleavage efficiency, especially when paired with a short-stalk NA, thereby augmenting virulence in both chickens and mice [43]. Another mutation observed in both isolates was the L216 substitution at the left edge of the RBS of the HA protein, which improves binding to α2,6-linked sialic acid receptors found in humans, potentially influencing host specificity [43, 44]. These mutations may, therefore, increase the virus’s potential for zoonotic transmission.

Most amino acid residues at the HA RBS in LSJ/Env/83 were conserved. However, in LSJ/Env/84, a V190A substitution was observed. This position plays a key role in determining binding affinity to sialyl-α2,6-linked receptors, with valine (V) conferring the highest affinity [45]. Furthermore, glycosylation sites and antigenic sites 1 and 2 in both isolates were consistent with those found in the China–Vietnam–Indonesia sublineage.

### Implications for public health and surveillance

LPAI strains have the capacity to act as genetic donors for other influenza viruses and may also directly infect humans, potentially leading to the emergence of novel human-infecting strains [46]. Since December 2015, 82 human cases of H9N2 infection have been reported globally, including two fatalities. Of these, 80 cases were from China, and two were from Cambodia (World Health Organization). In 2020, Dharmayanti *et al*. [21] documented a reassortant H9N2 virus that had incorporated genes from H5N1, one of the reassortants detected in live poultry markets.

**Figure 6:**
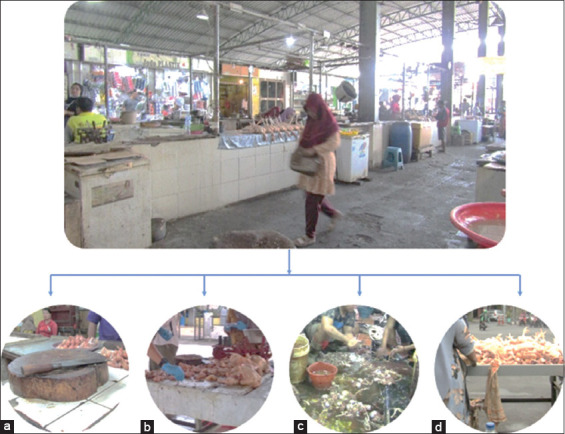
Critical areas of traditional markets and the possibility of human infection. (a) Cutting boards and knives; (b) display table and scales; (c) floor and wastewater; and (d) napkins.

The detection of the reassortant H9N2 virus in a traditional market in Lamongan Regency, East Java, highlights the urgent need for sustained surveillance to monitor the spread of H9N2 and other subtypes such as HxNx in high-risk market environments (Figure 6). The segmented genome of influenza viruses enables frequent mutations and reassortment through co-infection, wherein multiple subtypes infect a single host cell [47]. These reassortant viruses can give rise to new strains with either reduced or increased virulence and transmissibility, potentially broadening their host range to include mammals and humans - capabilities that may not have been previously observed by Rehman *et al*. [48] and Peacock *et al*. [49]. Despite this risk, the influenza virus remains an enveloped virus susceptible to alcohol and detergents [50], making disinfection a practical method for limiting environmental transmission.

The findings of this study reinforce the importance of routine monitoring to track the circulation of influenza A H9 and related subtypes. The detection of H9N2 isolates with molecular markers indicative of mammalian adaptation in environmental samples underscores the role of traditional markets as reservoirs and transmission hubs for zoonotic viruses. Continued surveillance in these settings is essential to detect emerging threats and inform public health responses.

## CONCLUSION

This study provides important insights into the environmental circulation and molecular characteristics of reassortant H9N2 AIV in traditional LBMs in East Java, Indonesia, during the COVID-19 pandemic. Among 156 environmental samples collected, 17 (10.9%) tested positive for influenza A, with three H9-positive samples originating from a single market in Lamongan Regency. Phylogenetic and molecular analyses of two isolates (LSJ/Env/83 and LSJ/Env/84) revealed that both were H9N2 reassortant viruses exhibiting key molecular markers - including Q226L, T160A, and S138A in the HA protein - associated with increased affinity for human-type receptors. Additional mutations in PB2, MP, and NS1 proteins supported their potential for enhanced replication, virulence, and cross-species transmission.

A major strength of this study lies in its combined use of environmental surveillance and genomic characterization, which enabled the identification of zoonotic markers directly from non-clinical samples in a high-risk setting. Furthermore, the integration of phylogenetic and molecular analyses provides robust evidence for the potential of LBMs to serve as reservoirs and amplification points for emerging AIV strains with zoonotic potential.

However, the study has some limitations. The environmental sampling was cross-sectional and limited to one province, which may not fully capture the broader spatial or temporal distribution of AIVs in Indonesia. In addition, the absence of viral isolation for all positive samples and lack of *in vivo* pathogenicity testing limits functional confirmation of the identified molecular markers. The study also did not assess concurrent infection or immune status in poultry or humans interacting within these markets.

Future research should focus on longitudinal, multi-provincial surveillance to monitor the dynamics of AIV transmission in LBMs, coupled with animal and human seroepidemiological studies. Functional assays, including receptor binding and pathogenicity studies in mammalian models, are warranted to further evaluate the zoonotic potential of identified reassortant strains. Strengthening integrated surveillance and implementing targeted interventions - such as periodic market closures for disinfection and enhanced biosecurity - are critical to mitigating the risk of future avian-origin influenza pandemics.

## AUTHORS’ CONTRIBUTIONS

NLPID, DN, and HN: Conception and design of the study, literature review, and manuscript drafting. NLPID and DN: Performed data analysis, figures and tables preparation, and software-related tasks. NLPID, DN, RI, and TS: Conducted fieldwork and laboratory investigations. All authors critically reviewed the manuscript for intellectual content, approved the final version, and agreed to be accountable for all aspects of the work.
